# Comparing CNNs and PLSr for estimating wheat organs biophysical variables using proximal sensing

**DOI:** 10.3389/fpls.2023.1204791

**Published:** 2023-11-20

**Authors:** Alexis Carlier, Sébastien Dandrifosse, Benjamin Dumont, Benoit Mercatoris

**Affiliations:** ^1^ Biosystems Dynamics and Exchanges, TERRA Teaching and Research Center, Gembloux Agro-Bio Tech, University of Liège, Gembloux, Belgium; ^2^ Plant Sciences, TERRA Teaching and Research Center, Gembloux Agro-Bio Tech, University of Liège, Gembloux, Belgium

**Keywords:** phenotyping, close-range sensing, wheat, CNN, biophysical variables, multi-task, PLSr

## Abstract

Estimation of biophysical vegetation variables is of interest for diverse applications, such as monitoring of crop growth and health or yield prediction. However, remote estimation of these variables remains challenging due to the inherent complexity of plant architecture, biology and surrounding environment, and the need for features engineering. Recent advancements in deep learning, particularly convolutional neural networks (CNN), offer promising solutions to address this challenge. Unfortunately, the limited availability of labeled data has hindered the exploration of CNNs for regression tasks, especially in the frame of crop phenotyping. In this study, the effectiveness of various CNN models in predicting wheat dry matter, nitrogen uptake, and nitrogen concentration from RGB and multispectral images taken from tillering to maturity was examined. To overcome the scarcity of labeled data, a training pipeline was devised. This pipeline involves transfer learning, pseudo-labeling of unlabeled data and temporal relationship correction. The results demonstrated that CNN models significantly benefit from the pseudolabeling method, while the machine learning approach employing a PLSr did not show comparable performance. Among the models evaluated, EfficientNetB4 achieved the highest accuracy for predicting above-ground biomass, with an R² value of 0.92. In contrast, Resnet50 demonstrated superior performance in predicting LAI, nitrogen uptake, and nitrogen concentration, with R² values of 0.82, 0.73, and 0.80, respectively. Moreover, the study explored multi-output models to predict the distribution of dry matter and nitrogen uptake between stem, inferior leaves, flag leaf, and ear. The findings indicate that CNNs hold promise as accessible and promising tools for phenotyping quantitative biophysical variables of crops. However, further research is required to harness their full potential.

## 1 Introduction

Biophysical vegetation variables are critical indicators of plant growth and health, providing essential information for understanding complex plant-environment interactions (Hawkesford and Riche, 2020; Lemaire and Ciampitti, 2020). Among these variables, Leaf Area Index (LAI), Aboveground Biomass (AGB), and Nitrogen Uptake (Nupt) stand out as key parameters that aid in crop monitoring and yield prediction. Additionally, they play a pivotal role in unraveling the underlying physiological processes that govern the intricate associations between final yield, genotype, and the surrounding environment. As concerns about climate change and the human food security continue to intensify, the accurate assessment of vegetation variables becomes increasingly crucial (Hickey et al., 2019). Timely and reliable information on crop growth and health can help optimize agricultural practices, enhance resource utilization, and help breeders and researchers improve crops.

Recent developments in phenotyping systems, utilizing multiple remote sensing platforms such as satellites, drones, and ground platforms equipped with various sensors (e.g., RGB, spectral data, thermal, LiDAR, etc…), have led to an improvement in the high-throughput and non-destructive screening of crops (Reynolds et al., 2020; Araus et al., 2022; Sun et al., 2022). These technologies have enabled the collection of large volumes of image data, facilitating the rapid, non-invasive, and detailed acquisition of plant phenotyping traits throughout the entire crop lifecycle (Verrelst et al., 2019). Remote sensing, which has lower spatial resolution, can capture the canopy in its entirety in a fast way. In contrast, proximal sensing provides more precise measurements at the organ level and might better handle the impact of unwanted factors (Deery et al., 2014). Ground-based phenotyping systems equipped with multiple sensors can acquire high-resolution data, facilitating improved identification of plant organs, diseases, or yellow and green plant parts (Carlier et al., 2022; Dandrifosse, 2022; Serouart et al., 2022; Tanner et al., 2022; Xu and Li, 2022). The integration of big data and machine/deep learning techniques further enhances the potential for precision phenotyping, enabling more accurate and efficient analyses of crop characteristics for enhanced agricultural management and breeding practices (Verrelst et al., 2019).

The assessment of such biophysical variables using remote sensing and proximal sensing methods requires a comprehensive understanding of agronomy, image and data analysis, given the inherent complexity of these traits and their susceptibility to various influencing factors. Usual methods for estimating AGB and LAI rely on crop architecture, vegetation indices, radiative transfer models, or a combination of these models (Tilly et al., 2015; Brocks and Bareth, 2018; Yue et al., 2019; Raj et al., 2021; Schiefer et al., 2021; Wan et al., 2021). Such methods are also widely used for assessing crop nitrogen status (Berger et al., 2020).

The algorithm pipeline commonly used in plant phenotyping comprises several stages, which involve feature extraction through image analysis methods, including color information collection, thresholding, edge detection, or/and pattern recognition. While these methods can be effective, their reliance on handcrafted features and hyperparameter tuning often results in a lack of robustness. This limitation becomes particularly evident when dealing with complex environmental conditions, such as the presence of soil, weeds, and biotic and abiotic stresses, as well as variations in plant characteristics like growth stage and canopy architecture. Thus, many phenotyping studies focus solely on local areas or specific agricultural practices, leading to limitations in the broader applicability and generalization of proposed models (Chao et al., 2019).

These challenges can lead to suboptimal performance and reduced accuracy in plant phenotyping tasks (Kamilaris and Prenafeta-Boldú, 2018; Nabwire et al., 2021). Yet, it becomes paramount to design studies that effectively capture the diversity present within crop populations and account for the variability of growing conditions. By doing so, we could unlock valuable insights into the intricate interactions shaping these biophysical variables, fostering more robust and adaptable solutions for the future (Hawkesford and Riche, 2020). To address these issues, researchers have been exploring the potential of deep learning and artificial intelligence techniques also in agricultural applications. These approaches have shown promising results in overcoming the limitations of traditional methods by automatically learning relevant features and adaptively adjusting to diverse conditions.

By leveraging advanced machine learning algorithms, such as deep neural networks and convolutional neural networks (CNNs), plant phenotyping can benefit from improved accuracy and generalization across varying scenarios (Singh et al., 2018; Kattenborn et al., 2021; Arya et al., 2022). These methods excel in handling complex datasets and can effectively capture intricate patterns and relationships in plant-related data. Additionally, they reduce the need for manual feature engineering and parameter tuning, leading to more efficient and reliable analyses. For instance, when predicting wheat biomass during early growth stages, CNNs demonstrated less susceptibility to plant density variations compared to alternative methods (Ma et al., 2019). Moreover, these innovative approaches enhance the ability to accurately estimate traits and unlock the extraction of more advanced parameters, such as crop growth rate, particularly when applied to time-series data (Buxbaum et al., 2022). Furthermore, their remarkable ability to solve highly complex patterns makes them ideal for multi-output purposes, enabling the production of multi-trait outputs using a single model (Pound et al., 2017; Nguyen et al., 2023).

The accessibility of ready-to-use libraries, datasets, and emerging methodologies like transfer learning has enabled the application of sophisticated algorithms to crop characterization. The ever-growing availability of neural networks architectures and hyperparameters can present a challenge when it comes to selecting or designing the most suitable architecture. While some authors have successfully created their own neural architectures that perform comparably to well-known ones in terms of accuracy (Li et al., 2021), it is still highly recommended to use established and widely recognized architectures. Nonetheless, ensuring the accuracy and robustness of these models is crucial, and their training and validation with large ground-truth datasets remain essential. This becomes particularly challenging when dealing with biophysical variables, such as AGB, which require a significant amount of human labor and destructive measurements to construct a dataset (Jiang and Li, 2020). This could explain why regression CNN is not yet widely adopted.

To address the need for data, several methods have been proposed to train robust models with a limited amount of labeled data. One approach is to use pre-trained models with transfer learning, which has been successful in estimating forage biomass (Castro et al., 2020; de Oliveira et al., 2021). However, when dealing with multispectral images, pre-trained models that are generally trained on RGB images may not perform well. Another approach is to use data augmentation to artificially increase the dataset size by applying transformations to the images. Advanced data augmentation methods, such as generative adversarial networks (GANs), have been used to improve wheat yield estimation (Zhang et al., 2022). Yet, phenotyping users often acquire large amounts of unlabeled data that still can be used to train a part of a CNN. Semi-supervised learning methods could be used to pre-trained the convolutional parts of CNN from unlabeled datasets (Zbontar et al., 2021). Additionally, one can predict labels for unlabeled data and subsequently insert them into the training dataset if they meet certain criteria; this technique is known as pseudo-labeling (Lee, 2013).

The use of CNNs in various domains has shown promise, and their potential in agriculture for regression purposes needs more investigation. The current study investigate the use of CNN for estimating biophysical variables such as AGB, LAI, nitrogen concentration, and nitrogen uptake from proximal images of wheat. While some studies have already shown some good examples of the use of CNNs for biomass or LAI prediction (Ma et al., 2019; Li et al., 2021; Sapkota et al., 2022; Schreiber et al., 2022; Zheng et al., 2022), many questions remain unanswered. These unanswered questions encompass identifying the optimal CNN architectures for achieving superior performance in estimating LAI, above-ground biomass, nitrogen uptake, and nitrogen concentration for wheat organs utilizing RGB and multispectral close-range images. Additionally, addressing the challenges related to insufficient training data and devising an effective training pipeline is imperative. Furthermore, there is a need to evaluate the effectiveness of multi-output models in assessing dry matter and nitrogen uptake partitioning, as well as nitrogen concentration partitioning in various wheat organs. Lastly, the best-performing CNN methods will be compared to a traditional machine learning approach, a Partial Least Squares regression (PLSr), using feature engineering.

## 2 Materials and methods

### 2.1 Experimental design

Data were acquired on winter wheat trials during four years in the Hesbaye area, Belgium (50 33’50” N and 4 42’00” E). Experimental microplots measuring 1.95 m × 6 m were sown with an inter-row spacing of 0.14 m, on homogeneous deep silt loamy soil in a temperate climate. The microplots were fertilized with 27% ammonium nitrate during the tillering, stem elongation and flag leaf stage corresponding to the BBCH 28, 30 and 39 growth stages, respectively. The trials were of two types: (i) trials testing different fertilization fractioning, noted as F and detailed in **Table S1** and in **Table S2**, (ii) trials composed of different fertilization fractioning combined with different fungicide application programs, noted as FP and detailed in **Table S3**. These abbreviations, along with the year of experimentation, are used in the trial names presented in **Table 1**.

**Table 1:** Field trial details.

Trial name	Cultivar	BBCH growth stages of samples	No. image acquisitions dates	Sensor	Sowing (grains/m^2^)	Sowing date (dd/mm/yyyy)
19-F	Safari	30, 32, 39, 65, 77, 89	11	RGB	250	23/10/2018
20-FP	LG Vertikal	39, 65, 89	11	RGB + MS	250	07/11/2019
20-F	Mentor	32, 39, 65, 75, 89	15	RGB + MS	250	05/11/2019
21-FP	LG Vertikal	39, 65, 89	16	RGB + MS	300	27/10/2020
21-F	Mentor	30, 32, 39, 65, 75, 89	15	RGB + MS	275	20/10/2020
22-F	Mentor	30, 65, 89	13	RGB + MS	300	28/10/2021
22-FP	Bennington	30, 32, 39, 65, 75, 89	17	RGB + MS	300	28/10/2021

In 2019, there was only one RGB camera, whereas the other years there were two RGB cameras and one multispectral (MS) camera.

#### 2.1.1 Reference measurements

Manual measurements were conducted on major phenological growth stages (**Table 1**), which mainly consisted of tillering, stem elongation, flag leaf, flowering, grain development, and maturity stages. The F trials involved five treatments, while the FP trials involved seven treatments, with three and four replicates conducted, respectively. Fresh AGB was sampled from the three central rows of the microplot over a length of 0.50 m. In the laboratory, the samples were manually separated into ear, stem, flag leaf (L1) and inferior leaves (Linf) groups. Each part was subsequently dried to determine the associated dry matter (DM) expressed in t/ha. The nitrogen concentration (%N) was then measured using the Dumas method, and nitrogen uptake (Nupt) was calculated by multiplying the DM by the corresponding %N, expressed in kgN/ha. Organs DM and Nuptake values of organs were expressed as relative values, representing the partitioning of DM and N uptake among each organ. These relative values indicate the proportion of each organ in relation to the total plant values. Additionally, the Nitrogen Nutrition Index (NNI) was computed using the traditional approach described in (Justes, 1994).

To determine LAI, plants were sampled by taking one row measuring 0.50 m in length. The leaves were separated from the stems, weighed, spread on a white paper using a transparent adhesive sheet, and scanned. An Otsu segmentation method was employed to isolate the leaves from the white background (Otsu, 1979). The leaf surface area was calculated by summing the areas of the scanned paper sheets multiplied by the proportion of pixels segmented as leaf. Since this protocol was time-consuming, only five microplots with contrasting fertilization were selected for manual LAI measurements at each collection date. These LAI values were correlated with the associated fresh masses by means of a linear regression to predict the LAI of the other microplots. Each correlation had a really high correlation above 0.9, thus validate this method as a reference.

#### 2.1.2 Image acquisitions

**Figure 1:**
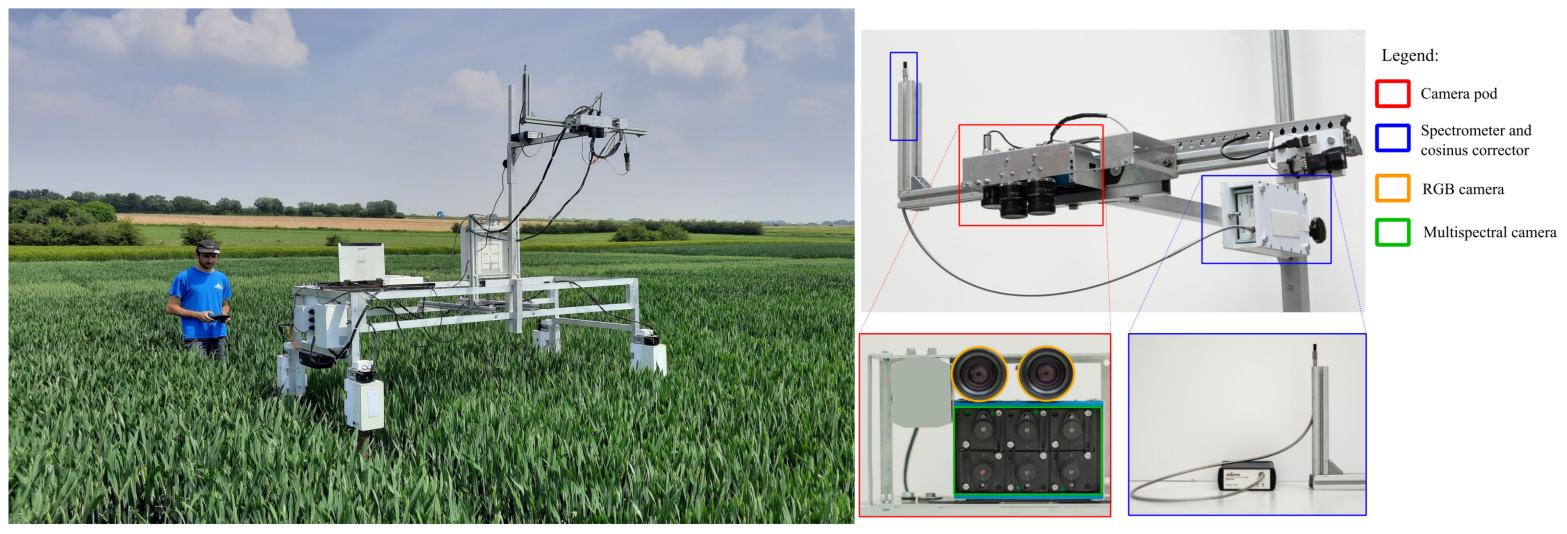
Experimental setup. A ground mobile platform (on the left) was equipped with a camera pod (on the right) comprising two high-resolution RGB cameras, a multispectral camera, and an incident light spectrometer, all positioned at a height of 1.6m above the canopy.

To capture nadir frames of wheat microplots, a phenotyping platform was designed (**Figure 1**). In 2019, a single RGB camera was utilized, while a sensor pod combining two types of cameras was employed in 2020, 2021, and 2022. The sensor pod comprised of two close-up RGB cameras dedicated to stereovision. These RGB cameras were GO-5000C-USB cameras from JAI A/S in Copenhagen, Denmark, and featured a 2560 × 2048 CMOS sensor. Additionally, a multispectral camera, the Micro-MCA from Tetracam Inc. in Gainesville, FL, USA, was used. It had six 1280 × 1024 pixel CMOS sensors, each of them equipped with narrow filter centered respectively at 490, 550, 680, 720, 800, and 900 nm. To avoid shadows from the rest of the platform in the images, both cameras were installed on a cantilever beam. The height of the cameras was adjusted at each acquisition date to maintain a consistent distance between the cameras and the top of the canopy. The height was about 1 m in 2019 and 1.6 m for the other years. Two to four images were taken per microplots for both cameras.

The RGB images were recorded using a color depth of 12-bit per pixel in 2019, 2020 and 2021, which were then converted to 8-bit to match the following algorithms. The multispectral grey scale images were converted from 10 to 8-bit, in accordance with the constructor recommendations. In 2019, the RGB camera auto-exposure algorithm was used. Then, a custom exposure algorithm was developed to limit the number of saturated pixels to less than 1%. The multispectral auto-exposure algorithm was based on a master-slaves principle. The 800 nm filter served as the master and its exposure time was determined automatically using the manufacturer algorithm. The exposure time of each slave filters was then defined as a ratio of the master time. These ratios were adjusted across the season to avoid saturated pixels.

**Figure 2:**
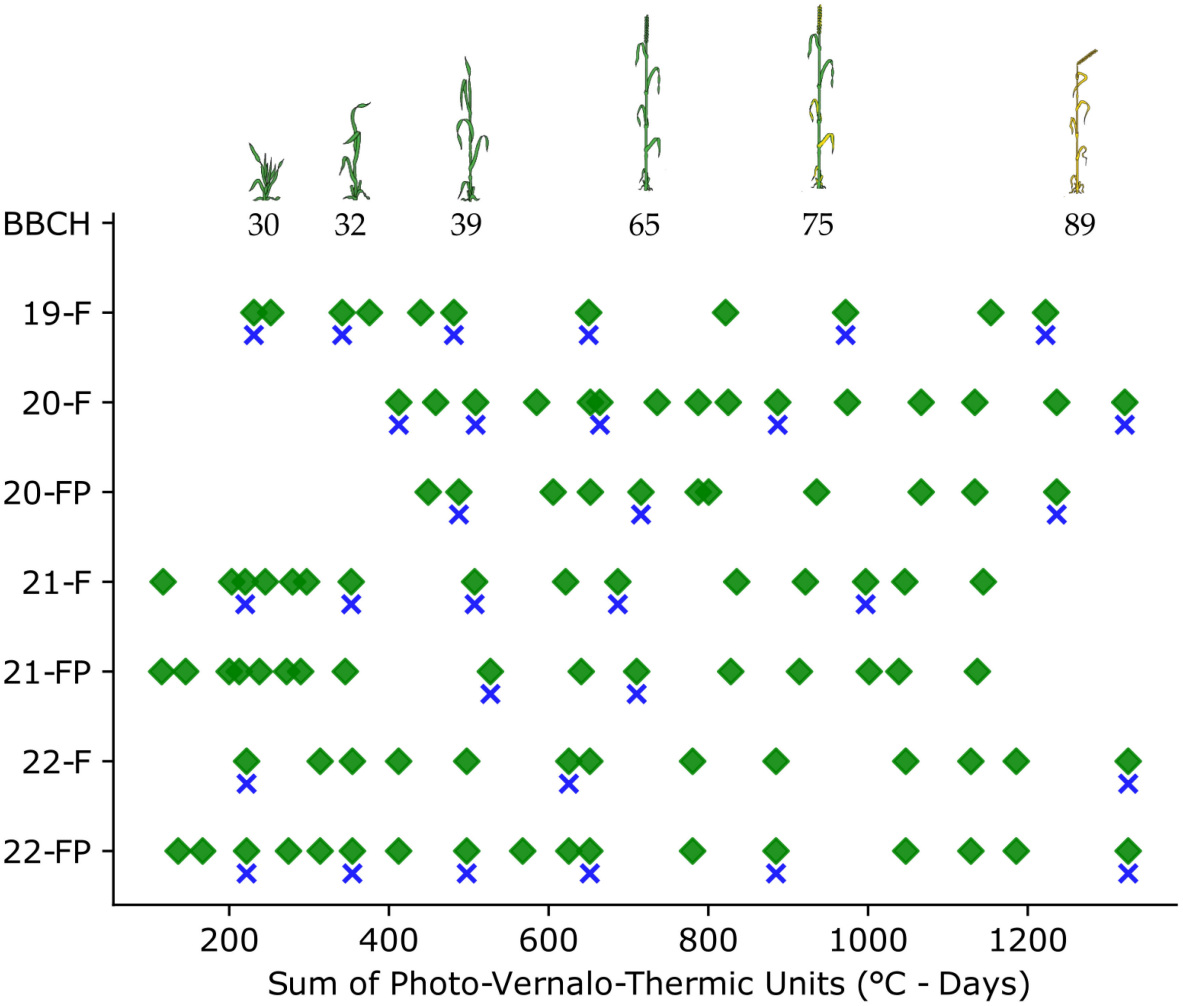
Overview of the data acquisitions during the cropping seasons. Green diamonds represent the image acquisitions, and the blue crosses the agronomic samples.

The cropping seasons were thoroughly covered, with multiple image acquisitions from tillering to maturity (**Figure 2**). Nevertheless, some unforeseen events occurred, such as the COVID-19 pandemic and a violent storm in 2021, which disrupted data acquisition.

The multispectral images underwent two pre-processing steps. The first step involved image registration to correct for shifts between the gray-scale images caused by the proximity to the canopy and the physical lenses gap. The considered method proposed by Dandrifosse et al. (2021) employs a b-spline approach to achieve pixel-wise alignment. The second step involved correcting the multispectral images for different light conditions during acquisition, using the method described by Dandrifosse et al. (2022). A laboratory calibration was performed to convert the digital numbers of the images to Bi-directional Reflectance Factor (BRF), known as reflectance, using an Incident Light Spectrometer, specifically an AvaSpec-ULS2048 from Avantes, Apeldoorn, The Netherlands.

### 2.2 Partial Least Squares regression approach

A conventional machine learning approach was tested to confront the CNN models presented below. As machine learning algorithms require relevant image features to be extracted, additional processes were applied after performing the pre-processing steps as described in the previous section. Firstly, a stereovision process was used to extract plant height information using the 95th percentile of the height map (Dandrifosse et al., 2020). Secondly, the plant ratio was computed as the proportion of plants in the scene, using a simple threshold method on the 800 nm image as detailed in Dandrifosse et al. (2022). Finally, twelve vegetation indices (see **Table S7**) were computed using the six BRFs.

A Partial Least Squares regression (PLSr) model was trained and validated using these twenty features for DM, %N and Nupt of the entire plant. It is worth noting that PLSr has previously exhibited good performance in analogous studies (Freitas Moreira et al., 2021). To fine-tune the model, a sequential backward feature selection approach was employed similar to (Song et al., 2022). This method involved generating all possible feature subsets of size n - 1, where n represents the total number of features. Each subset was rigorously assessed using a 5-fold cross-validation technique on the training dataset. The feature to be removed at each step was determined based on the subset’s performance, with the least contributing feature being eliminated. This iterative process continued until the maximum R² value was achieved. It is important to mention that the training data did not encompass the 2019 dataset, primarily due to the limited availability of only one RGB camera during that period. Furthermore, the efficacy of the pseudo-labeling strategy, as described in Section 2.3.3 was also explored for PLSr. This training was performed using the PLSr default parameters from the Scikit-Learn 1.3 Python library (Pedregosa et al., 2011).

### 2.3 CNN training

#### 2.3.1 Architecture

Three CNN architectures available in the python library Tensorflow 2.4. and Keras 2.4 were tested in this study. They were Resnet50 (He et al., 2015) and EfficientNetB0 and B4 (Tan and Le, 2020). They represent the actual state-of-the-art CNN models with different properties (i.e., architecture and number of parameters) and purposes. Resnet 50 was already used for biomass prediction by (Zheng et al., 2022) and EfficientNet is a cutting-edge neural network architecture with a remarkable ability to seamlessly scale from smaller to larger sizes while maintaining good efficiency.

The CNN architectures were customized to perform two tasks: (i) a single-output model to estimate LAI, DM, %N and Nupt of the whole plant respectively; and (ii) a multi-output model to estimate DM, %N, or Nupt of each wheat organ, also referred as partitioning model in the rest of this paper. Multi-output, also known as multi-task model have already been successfully used in phenotyping by (Nguyen et al., 2023) to predict a set of traits using a single model. Whereas a multivariate model deals with multiple dependent variables and aims to model their relationships, a multi-output model is a machine learning model designed to predict multiple output variables simultaneously. A linear activation function was considered for the last neuron of each single-output model. Regarding the multi-output models, four output neurons were considered, one for each organ. A linear activation function was used for the estimation of %N whereas the softmax activation function were used for the relative values of DM and Nupt, i.e., the proportion, in order to keep the values between 0 and 1. All models were initialized with weights from the ImageNet dataset (Deng et al., 2009).

The CNN architectures were originally designed for three-channel images, but the multispectral images used in this study had six channels. To accommodate this, a 2D convolutional layer with three filters and a kernel size of (1,1) was added at the beginning of each model when using multispectral images. It allowed to provide a three channels input required for the selected CNN models with pre-trained weights.

#### 2.3.2 Dataset configuration

The study used a dataset consisting of 1809 RGB images and 1391 multispectral images with their corresponding reference measurements. These numbers correspond to the multiplication of dates, samples, replicates, and images per microplots. Each image was associated with a specific combination of agronomic variables. From this dataset, two treatments from F trials (**Tables S1**, **S2**), and one treatment from FP trials (**Table S3**) were selected for the validation dataset that included 424 RGB images and 341 multispectral images.

In addition to the images acquired on the same days as the manual sampling, each trial was monitored continuously throughout the season, as illustrated in **Figure 2**. All those acquisitions yielded a dataset comprising 16 812 RGB images and 14 491 multispectral images. To prepare the data for the CNN models, some pre-processing steps were taken.

The first pre-processing step involved determining the image size, which is a trade-off between retaining as much information as possible and limiting the computing time and resources required. Additionally, when using pre-trained models, it is recommended to set the input image size to match the size used during initial training. Therefore, all images were resized to 224 x 224 for the ResNet50 and EfficientNetB0 models, and to 380 x 380 for the EfficientNetB4 model. It is worth noting that the images were previously cropped into a square to avoid distortion.

In addition to image resizing, the pixel scaling was also adjusted for each model. For the RGB images, pixel scaling was adapted according to the Keras documentation and the requirements of each model. For the multispectral images, Bi-directional Reflectance Factor (BRF) values were first normalized between 0 and 1. Next, the data was standardized based on the mean and standard deviation of the training dataset as advice by Tensorflow. To further enhance the dataset, data augmentation techniques, namely random flip up/down and right/left, were applied. These techniques increase the diversity of the dataset, which can improve the generalization performance of the models.

#### 2.3.3 Training pipeline

In the field of phenotyping, researchers often encounter a substantial amount of unlabeled data. However, these data hold untapped potential for enhancing the performance of machine learning models. In this study, a pseudo-labeling method was employed to leverage the unlabeled data effectively. Pseudo-labeling involves predicting the labels of unlabeled data using a model that demonstrates acceptable performance. These predicted labels, known as pseudo-labels, can then be incorporated into the training dataset, subject to a predefined confidence threshold. For classification tasks, this confidence threshold is based on class probabilities. Nevertheless, regression tasks utilize a linear activation function, leading to the absence of probabilities. To overcome this challenge, this research proposes a novel approach. The predicted biophysical variables from each microplot were plotted against time to generate a crop growth curve. This curve characterizes the growth pattern of the crop over time and can be harnessed to rectify the predicted values.

**Figure 3:**
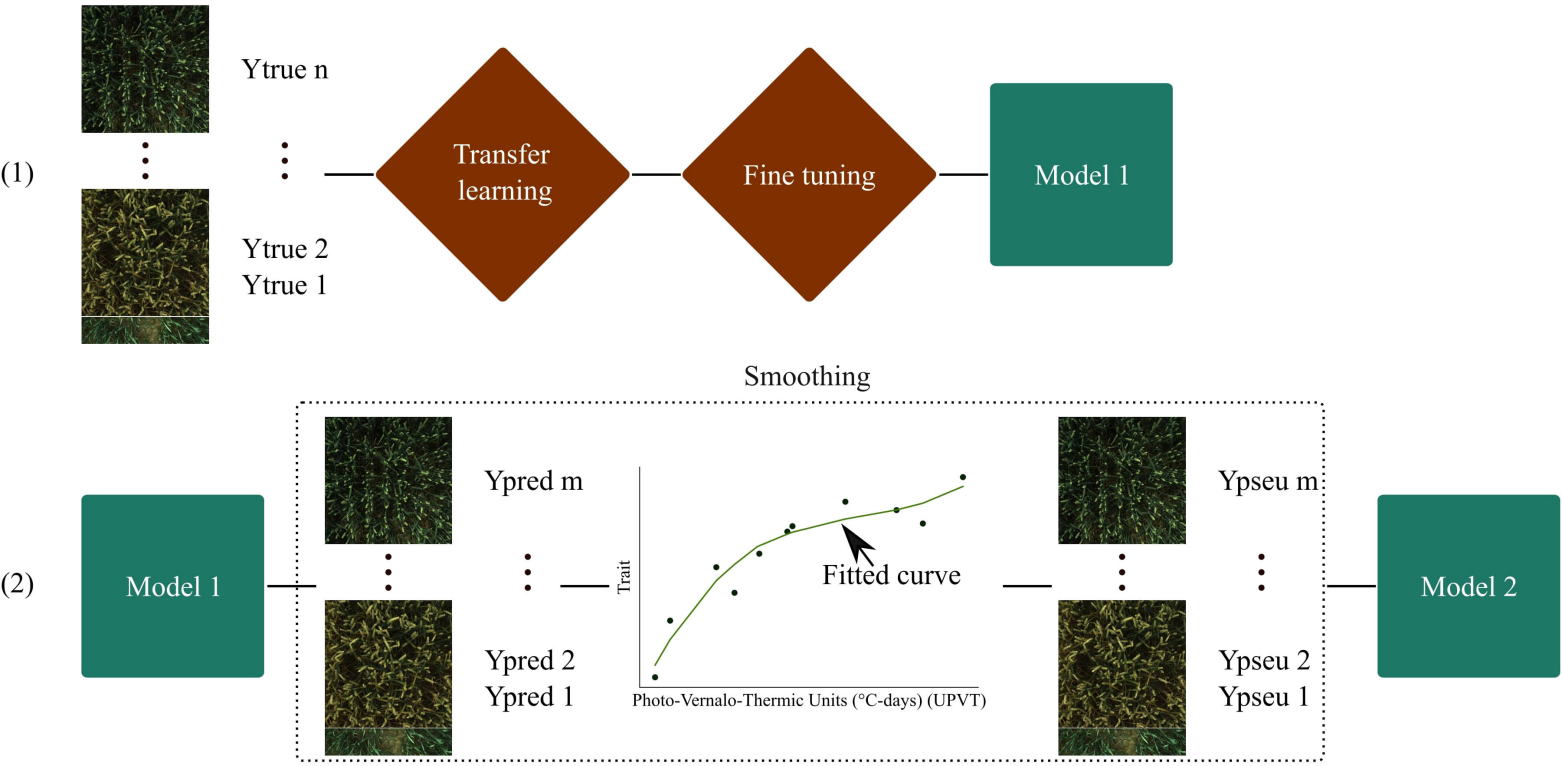
Proposed training pipeline. (1) is the training with transfer learning, and (2) is the training with pseudo-labels. Ytrue corresponds to the reference measurements. Ypred are predicted labels. A curve is fitted to provide the Ypseu which represent the corrected pseudo-labels. n and m correspond to the number of reference measurements and the total number of images respectively.

Based on this idea, a well-defined pipeline was constructed (see **Figure 3**). The pipeline entailed utilizing CNN models pre-trained on ImageNet through transfer learning. The initial training phase involved training the CNN models for 40 epochs with a learning rate of 1×10^−3^. During this process, only the last layer, specifically the linear dense layer, was trained, while keeping the remaining layers frozen.

Following this, a fine-tuning stage was conducted for 10 epochs, with a reduced learning rate of 1×10^−5^. During fine-tuning, the last convolutional layer block was unfrozen and retrained, leading to the creation of Model 1.

Next, Model 1 was utilized to generate predicted labels (Ypred) for the complete training dataset. These predicted labels were then plotted against the Photo-Vernalo-Thermic Units (°C-days) (Duchene et al., 2021). A cubic B-Spline for LAI and a cubic polynomial function for the other variables was fitted with a high smoothing condition. These curves are traditionally used in biophysical variables modeling (van Eeuwijk et al., 2019). Basic correction conditions were also implemented to help that fitting, such as setting organ values to 0 when they were not present at specific times. The outcome of this process yielded a fitted curve from which “corrected” pseudo-labels (Ypseu) could be extracted.

Last, pre-trained CNNs from ImageNet were trained on the corrected pseudo-labels (Ypseu) for 30 epochs, using a learning rate of 1×10^−5^. This resulted in the development of Model 2, which was thus trained on a much larger dataset compared to Model 1.

The Mean Square Error (MSE) loss function and Adam optimizer were used in all models. However, in the case of multi-output model for %N, the MSE calculation was limited to true labels above 0. This means that if an organ was not yet visible (e.g., the ear during tillering growth stage), the loss function did not take it into account, which prevented it from interfering with the loss function. Additionally, a weight was applied to the loss calculation when working with relative multi-output models. Specifically, the flag leaf pool weights were multiplied by twenty to ensure consistency with the order of magnitude of the other organ pools. This helped to balance the contributions of different organ pools and prevent one pool from dominating the loss calculation. All models were trained on an NVidia Tesla V100 GPUs.

To evaluate the performance of all models, two metrics were used: the determination coefficient (R²) and the root mean square error (RMSE).

## 3 Results

### 3.1 Variations of winter wheat biophysical variables

The descriptive statistics reveal significant variations in the four biophysical variables across the different growth stages: biomass ranging from 0.51 to 27.89 T/ha, LAI from 0.69 to 8.66, nitrogen concentration from 0.61 to 4.76%, and nitrogen uptake from 13.49 to 338.59 kg N/ha (**Table 2**). This wide variability in the datasets was attributed to diverse factors, including variations in growing stages, repeated measurements over multiple years, and heterogeneous treatments, particularly variations in nitrogen inputs. The analysis, specifically employing ANOVA, indicates that most of these biophysical variables exhibit significant differences among treatments (**Table S4**). Both the training and validation datasets exhibit similar statistics, which validates the appropriateness of the dataset splitting method. Furthermore, correlations between these variables were examined, and the results show that biomass demonstrated a Pearson correlation of -0.27, -0.71, and 0.87 with LAI, nitrogen concentration, and nitrogen uptake, respectively. The correlation between nitrogen concentration and nitrogen uptake was found to be -0.44.

**Table 2:** Descriptive statistics of dry matter (T/ha), LAI, N concentration (%) and N uptake (kg N/ha).

Dataset	Statistic	Dry matter (T/ha)	LAI	N concentration (%)	N uptake (kg N/ha)
Training	mean std min max	10.44 6.15 0.51 23.07	3.40 1.65 0.72 8.66	1.81 0.85 0.61 4.76	152.31 77.37 13.49 338.59
Validation	mean std min max	10.32 6.00 0.51 27.89	3.28 1.45 0.69 7.35	1.76 0.80 0.87 4.09	145.21 66.86 16.26 307.14

### 3.2 Plant biophysical variable modeling

This study evaluated various models for predicting plant biophysical variables. The EfficientNetB4 model trained on pseudo-labels demonstrated the highest performance for DM, achieving an R² of 0.92 and a low RMSE of 1.50 on the validation dataset (**Table 3**). In contrast, the PLSr model had an R² of 0.77 and a higher RMSE of 2.58, indicating weaker predictive ability.

**Table 3:** Model performances for DM of the plant.

Model	Data	RMSE train	R² train	RMSE val	R² val
EfficientNetB0	Ytrue	1.76	0.91	2.11	0.83
EfficientNetB0	Ypseu	0.91	0.95	1.66	0.91
EfficientNetB4	Ytrue	1.38	0.93	1.89	0.86
EfficientNetB4	Ypseu	1.09	0.96	1.50	**0.92**
Resnet50	Ytrue	0.78	0.98	1.87	0.89
Resnet50	Ypseu	1.05	0.97	1.64	0.90
PLSr	Ytrue	2.49	0.80	2.55	0.78
PLSr	Ypseu	1.32	0.92	2.58	0.77

Regarding LAI, the ResNet50 model trained on pseudo-labels yielded the best R² of 0.82 (**Table 4**). For nitrogen concentration prediction using multispectral images, the ResNet50 model achieved an R² of 0.80 (**Table 5**) and an R² of 0.73 for Nitrogen uptake (**Table 6**).

**Table 4:** Model performances for LAI.

Model	Data	RMSE train	R² train	RMSE val	R² val
EfficientNetB0	Ytrue	1.06	0.69	1.18	0.57
EfficientNetB0	Ypseu	0.72	0.86	0.80	0.80
EfficientNetB4	Ytrue	0.68	0.86	0.78	0.80
EfficientNetB4	Ypseu	0.67	0.87	0.78	0.81
Resnet50	Ytrue	0.27	0.98	0.79	0.79
Resnet50	Ypseu	0.66	0.98	0.78	**0.82**
PLSr	Ytrue	0.77	0.75	0.83	0.75
PLSr	Ypseu	0.28	0.95	0.91	0.69

**Table 5:** Model performances for %N of the plant.

Model	Data	RMSE train	R² train	RMSE val	R² val
EfficientNetB0	Ytrue	0.37	0.74	0.36	0.72
EfficientNetB0	Ypseu	0.24	0.90	0.30	0.79
EfficientNetB4	Ytrue	0.33	0.75	0.32	0.55
EfficientNetB4	Ypseu	0.23	0.89	0.31	0.73
ResNet50	Ytrue	0.14	0.97	0.32	0.78
ResNet50	Ypseu	0.24	0.90	0.30	**0.80**
PLSr	Ytrue	0.49	0.59	0.43	0.54
PLSr	Ypseu	0.20	0.88	0.44	0.51

**Table 6:** Model performances for Nupt of the plant.

Model	Data	RMSE train	R² train	RMSE val	R² val
EfficientNetB0	Ytrue	42.42	0.65	37.97	0.66
EfficientNetB0	Ypseu	28.84	0.84	34.38	0.72
EfficientNetB4	Ytrue	37.46	0.70	43.39	0.47
EfficientNetB4	Ypseu	25.33	0.87	34.05	0.69
ResNet50	Ytrue	14.24	0.96	37.27	0.68
ResNet50	Ypseu	26.69	0.86	33.89	**0.73**
PLSr	Ytrue	39.72	0.65	39.89	0.70
PLSr	Ypseu	17.78	0.88	42.81	0.66

The other CNN models investigated in this study exhibited robust and comparable performance levels when subjected to the pseudo-labeling pipeline during training. The utilization of pseudo-labels played a pivotal role in mitigating disparities between the outcomes observed on the validation and training datasets. It is worth noting that the PLSr model did not yield any discernible advantages from the pseudo-labeling technique, consistently falling short of the CNN models in terms of performance. One noteworthy observation is that this pseudo-labeling method appeared to exacerbate the disparities between the performance of the training and validation sets. Furthermore, the results of the backward feature selection analysis, as depicted in **Figures S1** to **S4**, indicated that the augmentation of the dataset via this approach led to an increased requirement for features to achieve optimal performance levels.

**Figure 4:**
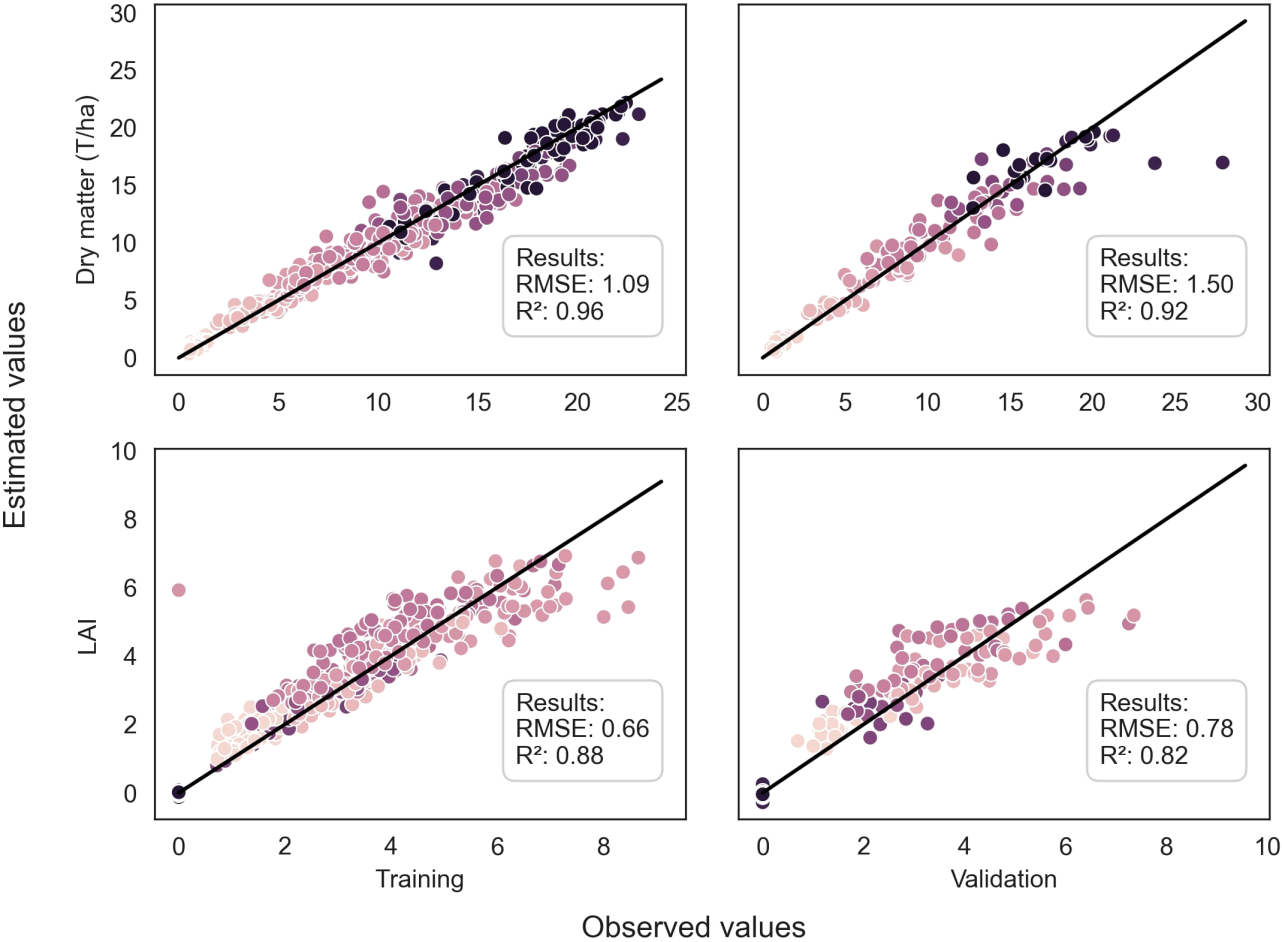
Comparison between observed and predicted values of DM of the whole plant and LAI for both training and validation datasets, using the EfficientNetB4 model for DM and the ResNet50 model for LAI. The dots are color-coded according to the stages in the season, with darker dots indicating later stages. The dark line represents the 1:1 line.

**Figure 5:**
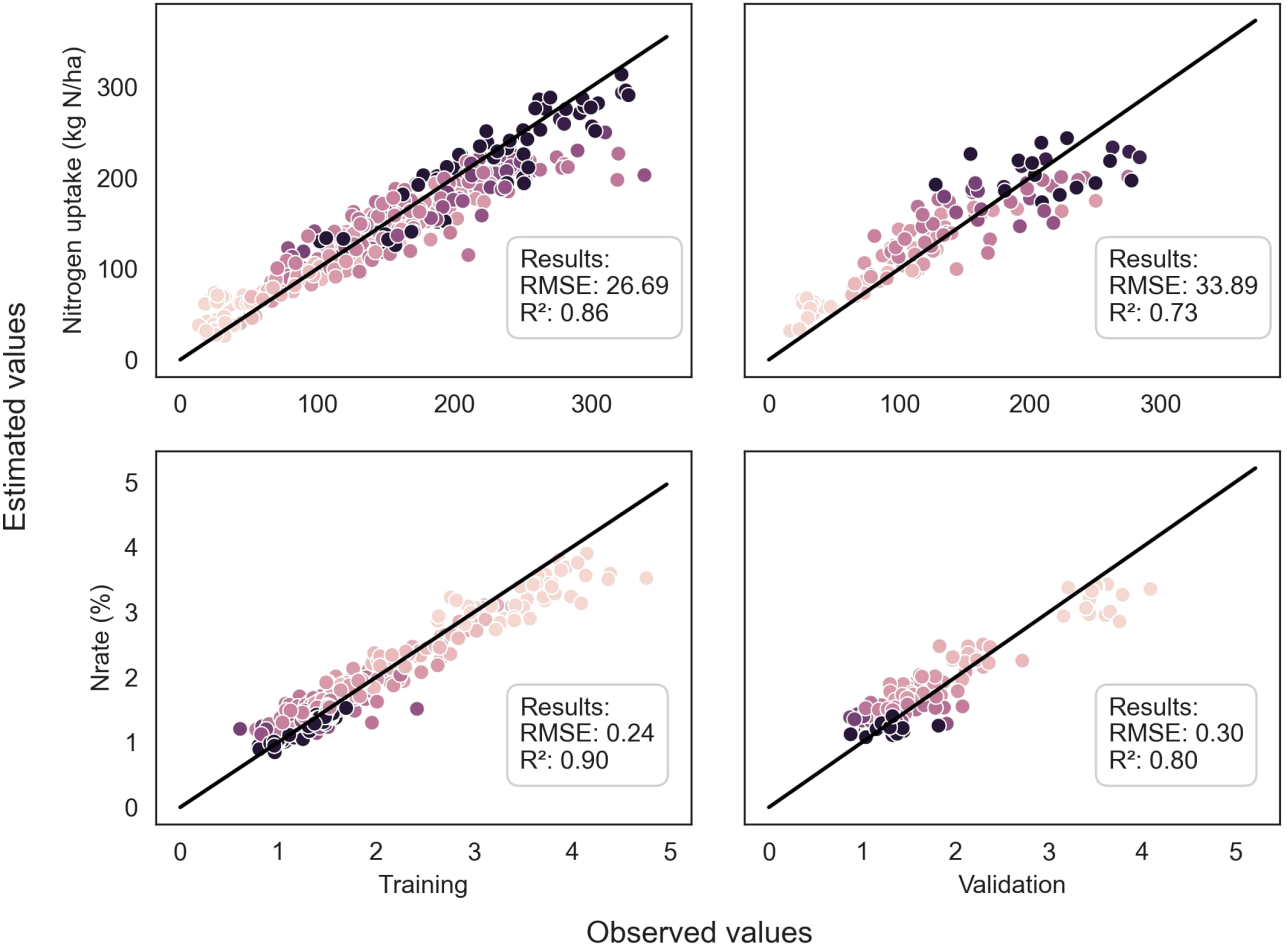
Comparison between observed and predicted values of %N and Nupt of the whole plant for both training and validation datasets, using the ResNet50 model. The dots are color-coded according to the stages in the season, with darker dots indicating later stages. The dark line represents the 1:1 line.

Throughout the growing season, the models successfully assessed the variables, as evidenced by **Figures 4** and **5**. However, there were some outliers that significantly deviated from the ideal 1:1 relationship between predicted and true values. Additionally, a saturation effect was observed, where the models struggled to accurately predict the maximum values of each variable, leading to a lack of detail in certain growing seasons. These observations provide valuable insights for further refining the modeling approach and improving predictive accuracy.

### 3.3 Organs biophysical variable modeling

The utilization of multi-output models yielded diverse outcomes regarding the proportion of dry matter and nitrogen uptake, as indicated in **Table S5**. **Table 7** displays the performance of the multiplication of both the single output models and the multi-output models for dry matter and nitrogen uptake, and solely the multi-output model for nitrogen concentration.

**Table 7:** R² of the different multi-outputs models to predict nitrogen uptake, dry matter and nitrogen concentration of each organ.

Model	Data	Dataset	Nuptake				DM				%N			
			Stem	Linf	L1	Ear	Stem	Linf	L1	Ear	Stem	Linf	L1	Ear
EfficienNetB0	Ypseu Ytrue	train train	0.84 0.72	0.78 0.67	0.77 0.7	0.92 0.91	0.91 0.90	0.49 0.49	0.08 0.17	0.97 0.96	0.71 0.60	0.86 0.75	-0.08 0.60	-2.18 -1.09
EfficienNetB4	Ypseu Ytrue	train train	0.54 0.6	0.02 0.48	0.65 -0.47	0.75 0.7	0.90 0.92	0.53 0.65	-0.11 0.57	0.96 0.97	0.67 0.38	0.75 0.56	-0.10 0.10	-1.95 -2.46
ResNet50	Ypseu Ytrue	train train	0.8 0.84	0.75 0.78	0.76 0.8	0.93 0.96	0.88 0.93	0.51 0.76	0.15 0.67	0.94 0.98	0.73 0.86	0.87 0.93	-0.05 0.95	-2.10 0.52
EfficienNetB0	Ypseu Ytrue	val val	0.7 0.63	0.59 0.47	0.69 0.54	0.86 0.86	0.83 0.82	0.28 0.34	-0.09 -0.16	0.95 0.93	0.54 0.47	0.84 0.69	-0.43 0.58	-2.64 -1.72
EfficienNetB4	Ypseu Ytrue	val val	0.51 0.41	-0.2 0.3	0.52 -1.66	0.66 0.65	0.83 0.84	0.38 0.55	-0.15 0.14	0.94 0.94	0.59 0.10	0.73 0.55	-0.46 0.13	-2.11 -3.91
ResNet50	Ypseu Ytrue	val val	0.64 0.67	0.52 0.56	0.64 0.66	0.85 0.86	0.87 0.87	0.57 0.62	0.22 0.38	0.94 0.94	0.48 0.50	0.84 0.76	-0.45 0.69	-3.17 -1.07

Among the models evaluated, EfficientNetB0 demonstrated superior performance for predicting nitrogen uptake, achieving commendable R² values of 0.7, 0.59, 0.69, and 0.86 for stem, inferior leaves, flag leaf, and ear, respectively. ResNet50 exhibited R² values of 0.87, 0.62, 0.38, and 0.94 for dry matter, and 0.50, 0.76, 0.69, and -1.07 for nitrogen concentration, indicating its effectiveness in certain cases.

Analyzing individual organs, the ear and stems exhibited higher prediction accuracy by the models, while the flag leaf showed comparatively poorer prediction, depending on the specific model employed. Concerning the %N models, the stem and inferior leaf pools were accurately predicted, but the prediction performance for the ear was notably inadequate.

Interestingly, while the pseudo-labeling method led to reduced performance for the multi-output models (**Table S5**), its combination with the single output models, which significantly benefit from pseudo-labels, did not have a substantial impact on the prediction of DM and %N for each organ. This suggests that the pseudo-labeling approach is effective in enhancing the single output models but may require further optimization for multi-output models.

**Figure 6:**
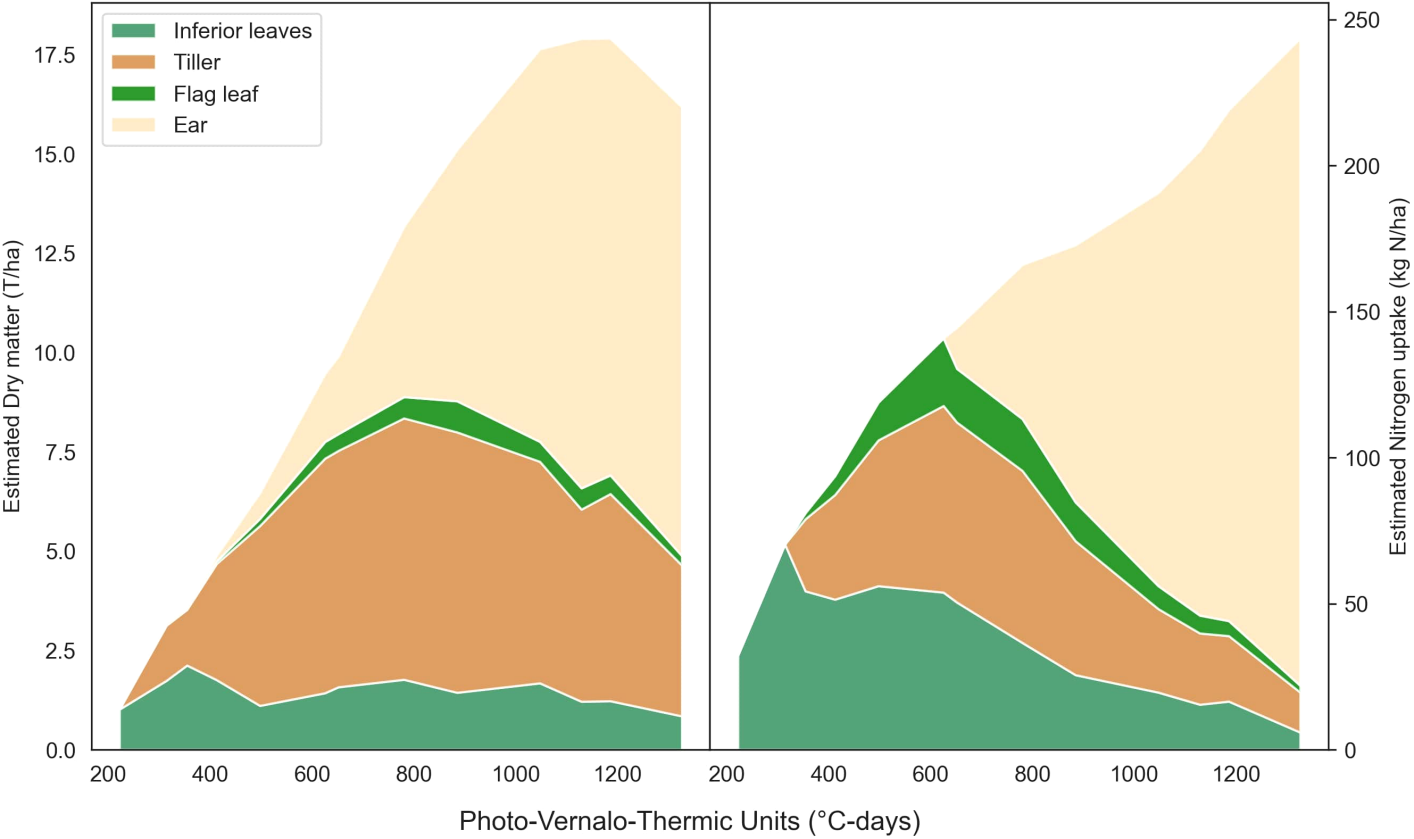
Predicted partitioning of dry matter and nitrogen uptake throughout the season for a microplot from the 22-F trial. This results from the use of the multi-output ResNet50 (**Table S5**) multiplied by the single output ResNet50 (**Table 3**).

The **Figure 6** presents the predicted partitioning of wheat dry matter and nitrogen uptake over the growing season for a single microplot. It offers a nice alternative to provide valuable insight about the partitioning of the matter within the plant. Moreover, both RGB and multispectral models successfully detected the emergence of new organs, such as the flag leaf and ear. Notably, the dry matter model showed an earlier appearance of ears compared to the nitrogen uptake model in this specific example.

## 4 Discussion

### 4.1 Convolutional neural networks as an effective approach for predicting biophysical variables

This study presents a comprehensive investigation into the potential of recent CNNs in accurately predicting biophysical vegetation variables, such as dry matter, leaf area index, and nitrogen uptake and concentration. The research demonstrate that our CNN-based approach stands as one of the most advanced methods for this task, even though direct performance comparisons with prior studies are hindered by the limited availability of benchmark datasets.

In this study, CNN models outperformed a PLSr approach, consistent with previous research findings (Ma et al., 2019; Castro et al., 2020). Thus, CNN stands out as a potent tool in this context due to its ability to autonomously extract features, eliminating the need for manual feature extraction. It demonstrates remarkable adaptability in handling the evolving features of crops throughout the growing season, including changes in physiology and color. This adaptability negates the necessity for fine-tuning models to specific growth stages or cultivars, as highlighted by previous machine learning research (Yue et al., 2019). These studies commonly adopt a strategy of employing one model for each growth stage, alongside a single overarching model that typically yields less satisfactory results (Wang et al., 2022a). Nevertheless, it would remain intriguing to explore the performance of the presented CNN models on new cultivars, which may exhibit distinct characteristics.

Moreover, the results pertaining to nitrogen concentration are particularly intriguing. One might expect that CNNs would prioritize features related to plant architecture, which would be more closely associated with nitrogen uptake. However, the observed medium correlation (-0.44) between nitrogen concentration and uptake tends to limit this assumption, suggesting that the CNNs might have also identified some kind of vegetation indices contributing to the predictions. Despite these remarkable outcomes, interpreting the specific features extracted by CNNs remains challenging. To enhance our understanding of the underlying mechanisms and improve model interpretability, ongoing research is dedicated to developing techniques for explaining CNN predictions. One such approach, Grad-CAM (Selvaraju et al., 2020), shows promise in providing insights into the regions of the image that significantly influence the model’s decisions.

Among the CNN architectures explored, ResNet50 exhibited high performance, consistent with similar studies (Castro et al., 2020). Notably, EfficientNet also yielded promising results, especially for DM prediction of the entire plant. However, it is worth considering that the advantage of EfficientNetB4 might be attributed to its capacity to capture finer details in larger images. Interestingly, recent research has shown that performance gains may saturate beyond a certain image size (Li et al., 2021). This behavior could be dependent on the architecture, as EfficientNet is explicitly designed for scalable optimization on specific datasets (Tan and Le, 2020).

In contrast, the machine learning approach utilizing PLSr and feature fusion from the multi-sensor system consistently delivered inferior performance when compared to CNN models. Nevertheless, it is important to underscore that this method still achieved commendable results, boasting an R² value exceeding 0.6, which aligns with the findings reported in Yue et al. (2019).

An intriguing aspect arising from the backward feature selection analysis was the observed increase in the number of selected features between Ytrue and Ypseu, signifying the heightened demand for features in constructing models with a larger dataset (See **Supplementary Material**). Additionally, both sets of features exhibited substantial similarities, affirming their efficacy in modeling agronomical parameters. Among these features, plant height emerged as the most frequently utilized, followed by plant ratio and MCARI index. Furthermore, it is noteworthy that DM and Nuptake shared three out of four features, a logical outcome given that Nuptake was derived from DM. Other features selected for nitrogen-related analysis included well-established indices such as MCARI, mNDB, and GR.

However, it is essential to exercise caution when drawing overarching conclusions solely based on this method. Notably, the selection of these features can be intricate, as they may exhibit seasonal variations, as documented in (Yue et al., 2019; Wang et al., 2022a, b). It is conceivable that more advanced methods may yield superior results, as suggested in (Wang et al., 2021).

### 4.2 The significance of the amount of ground truth data in deep learning for regression of biophysical variables

Deep learning techniques, especially in regression tasks involving biophysical variables, encounter a substantial challenge due to the scarcity of sufficient training datasets. The limited availability of labeled data necessitates the development of innovative approaches to overcome this issue. A training pipeline was devised in this study, which capitalizes on the abundance of unlabeled data commonly found in highthroughput phenotyping installations. This method represents a practical approach to leverage unlabeled data, leading to optimized performance of CNN models in phenotyping applications.

The pseudo-labeling method emerged as an effective strategy to mitigate overfitting of the model. As a result, the performance gap between the training and validation datasets was reduced, signifying enhanced generalization. To perform such data correction, a polynomial cubic curve was chosen for its simplicity in representing biophysical curves and ease of fitting. Finer curves more related to plant growth pattern, such as P-splines or logistic curves, could have been used, but the fitting process may prove difficult (van Eeuwijk et al., 2019). These finer curves often require more frequent measurements (one to two per week) for accurate fitting (Roth et al., 2020), a frequency that our data did not meet. To address potential bias, correcting conditions were introduced, particularly essential for organ models. For example, when an organ was absent at a specific time (t), the corresponding pseudo-label was set to 0, a correction that, while seemingly straightforward, significantly contributed to the accuracy of representations. The effectiveness of traditional machine learning might also be a good option to generate pseudo-labels in the case of fewer ground truth data.

During the research, we also examined more advanced data augmentation techniques, such as 90° rotation and color space transformations without success. It is crucial to exercise caution when employing such methods, as their indiscriminate application may adversely affect model performance, as observed in certain models in (Castro et al., 2020). Conversely, (Ma et al., 2019) reported clear performance improvements with these methods. The discrepancy in results may be attributed to the risk of the model becoming overly reliant on specific features, such as wheat lines in the case of image rotation. Hence, prudent consideration of data augmentation is warranted based on the specific characteristics of the dataset and model.

### 4.3 Limitations and perspectives

An effective approach for evaluating model performance is to combine their predictions into a single other variable. In this study, we used DM and %N of the plant, predicted from their respective models, to calculate the Nitrogen Nutrition Index (NNI). The R² values for the training and validation datasets were 0.71 and 0.33, respectively, suggesting the potential utility of this method for measuring NNI as well. Although the dataset contains a substantial amount of heterogeneous ground truth data, the performance of the models may raise questions due to its limited size in terms of crop architecture and color, which only includes a few genotypes. The observed patterns in predicted values in **Figures 5** and **4** appeared scattered, resembling a cloud rather than forming a clear line, and some outliers were evident, indicating room for improvement in the models. Overfitting was also observed, particularly with ResNet50, which frequently achieved R² values above 0.95 for the training dataset. To address this, a prudent approach would be to initially use small architectures and acquire more data. Despite the need for improvements concerning trait saturation and accuracy within specific growth stages **Figure 4**, the models’ potential is significant. They can be employed to compute advanced traits, such as growth rate and spot ideotypes using temporal curves, as demonstrated in a recent study (Roth et al., 2022).

By leveraging diverse and large-scale datasets, CNNs can yield more robust and precise models, reducing the need for heavily relying on study-specific feature engineering. Therefore, the phenotyping community should prioritize the development of extensive and well-annotated datasets for essential phenotyping challenges, such as the Global Wheat Head Detection (GWHD) dataset (David et al., 2021). Additionally, exploring alternative solutions, such as self-supervised learning (Zbontar et al., 2021) or generating synthetic data using Functional-Structural Plant Models (FSPM) (Gao et al., 2023), can further enhance model training and performance.

Research on the allocation of major plant elements, such as sink/source regulation processes and their relationship with grain nitrogen content, heavily relies on dry matter and nitrogen uptake partitioning (Martre et al., 2003; Gaju et al., 2014). The multi-output models proposed in this study have shown promising results (**Table 7**), with good performance in most cases. However, certain organs exhibited poor performance, such as %N of the ear, which may be attributed to the lack of visible traits that could account for it, like a greener ear. The subpar performance of DM and Nupt for flag leaf could be mainly attributed to the multi-output proportion model’s poor performance for this organ (**Table S5**), despite assigning it a higher weight in the loss function. Additional images specifically featuring flag leaves might be needed to improve its representation, as the ear rapidly develops behind them.

This multi-output model exemplifies the potential of such approaches for plant phenotyping. While this study employed a simple approach by sharing a common loss function, the benefits of multi-output learning can be substantial. For instance, a single model assessing both dry matter and leaf area index can significantly reduce computational costs and processing time, while maintaining high accuracy for both tasks. In fact, when tasks share complementary information, they can act as regularizers for each other, enhancing prediction performance (Standley et al., 2020). However, combining complex associations between tasks, such as classification and regression tasks, requires careful consideration of model architecture, loss function, and training strategy to achieve optimal performance. Ongoing research in this area is actively being pursued (Vafaeikia et al., 2020; Vandenhende et al., 2020).

The ability of the models to autonomously discover the appearance of new organs, such as ears and flag leaves, is particularly intriguing and opens exciting new research avenues too. This suggests the feasibility of developing growth stage estimation models per RGB image in a similar manner. Such models could be further utilized for various purposes, such as optimizing crop models (Yang et al., 2021).

## 5 Conclusions

In this study, a robust training pipeline that leverages unlabeled data through the innovative combination of pseudo-labeling and temporal relationship correction were developed and implemented. The results demonstrate the significant advantages of employing CNN models over a PLSr approach, as they achieve superior performance without the need for labor-intensive feature engineering. Notably, EfficientNetB4 was better in predicting above-ground biomass, while ResNet50 exhibited superior performances in predicting LAI, nitrogen uptake, and nitrogen concentration. Additionally, our exploration of multi-output models provided valuable insights into the distribution of dry matter and nitrogen uptake among different plant organs, enriching our understanding of plant biophysical characteristics.

While CNN models show great promise, it is evident that further investigation is required to fully unlock their potential. This research effectively demonstrates the capabilities of CNNs in predicting biophysical vegetation variables and offers valuable insights into addressing limitations and future perspectives in plant phenotyping. Moving forward, data sharing within the phenotyping community will be critical to optimize model performance. Access to large and diverse datasets, such as the Global Wheat Head Detection dataset, is indispensable for advancing phenotyping research and enhancing the performances of CNN models. By fostering data sharing and continued research efforts, CNNs can continue to revolutionize plant phenotyping and make profound contributions to agricultural and environmental sciences.

## Data availability statement

The raw data supporting the conclusions of this article will be made available by the authors, without undue reservation.

## Author contributions

AC and SD performed experiments and data collection; AC build the models, performed the statistical analysis, interpreted the results, and prepared the first draft. BD and BM contributed to the result interpretation and supervised the project. All authors revised the manuscript. All authors contributed to the article and approved the submitted version.

## Supplementary material

Supplementary Table 1 Fertilization trial (F) in 2020, 2021 and 2022.

Supplementary Table 2 Fertilization trial (F) in 2019.

Supplementary Table 3 Trials composed of different fertilization fractioning combined with different fungicide application programs (FP) in 2020, 2021 and 2022.

Supplementary Table 4 Dry matter, LAI, nitrogen concentration and nitrogen uptake of the whole plant used in this study. Data are means +/- the standard deviation (n=3 or 4 repetitions). Significance of treatment effects were analyzed using a one-way ANOVA (*: P ≤ 0.05; **: P ≤ 0.01; **: P ≤ 0.001).

Supplementary Table 5 R² of the different models to predict DM and Nupt proportion of each organ.

Supplementary Table 6 Pearson correlation table of the agronomic data

Supplementary Table 7 Vegetation indices selected in this study.

Supplementary Figure 1 Backward feature selection with PLSr for DM Estimation: On the left, selected features for estimating DM from Ytrue include Plant Ratio, 95th Percentile of Height, MCARI, and BRF 490. On the right, the selected features for estimating DM from Ypseu comprise Plant Ratio, 95th Percentile of Height, MCARI, and BRF 550.

Supplementary Figure 2 Backward feature selection with PLSr for LAI Estimation: On the left, selected features fo estimating LAI from Ytrue include SR, GNDVI, MCARI, CIgree, BRF 900 and BRF 720. On the right, the selected features for estimating LAI from Ypseu comprise NDRE, SR, GNDVI, CIgreen, CIrede and BRF 550.

Supplementary Figure 3 Backward feature selection with PLSr for Nuptake Estimation: On the left, selected features for estimating Nuptake from Ytrue include Plant Ratio, 95th percentile of height and MCARI. On the right, the selected features for estimating Nuptake from Ypseu comprise Plant Ratio, 95th percentile of height, GR, MCARI, mNDB and BRF 550.

Supplementary Figure 4 Backward feature selection with PLSr for Nrate Estimation: On the left, selected features for estimating Nrate from Ytrue include 95th percentile of height and MCARI. On the right, the selected features for estimating Nrate from Ypseu comprise 95th percentile of height, GR and mNDB.
