# Supplementary material for: Comparing CNNs and PLSr for estimating wheat organs biophysical variables using proximal sensing
Source: Front Plant Sci. 2023 Nov 20;14:1204791. doi: 10.3389/fpls.2023.1204791 (PMC10694231; doi:10.3389/fpls.2023.1204791)
Supplement: Supplementary Table 1 — Fertilization trial (F) in 2020, 2021 and 2022. [file Presentation_1.pdf]

Comparing CNNs and PLSr for estimating wheat organs biophysical variables using proximal sensing

Alexis Carlier[1], **Sébastien Dandrifosse**[1], **Benjamin Dumont**[2] **and Benoît Mercatoris**[1,*]

[1] *Biosystems Dynamics and Exchanges, TERRA Teaching and Research Center, Gembloux Agro-Bio Tech, University of Liège, 5030 Gembloux, Belgium*
[2] *Plant Sciences, TERRA Teaching and Research Center, Gembloux Agro-Bio Tech, University of Liège, 5030 Gembloux, Belgium*

Correspondence*:
Corresponding Author
benoit.mercatoris@uliege.be

SUPPLEMENTARY MATERIALS

DATA AVAILABILITY STATEMENT

Data and codes mentioned in this study are available on request from the corresponding author.

Table S1. Fertilization trial (F) in 2020, 2021 and 2022.

Nitrogen inputs (kgN/ha) at:			Total nitrogen inputs (kg.ha-1)	Destructive measurements	Train/Val
Tillering	Stem elongation	Flag leaf			
0	0	0	0	✓	Train
30	30	30	90	✓	Val
40	40	40	120		
50	40	55	145		
60	60	60	180	✓	Train
80	40	60	180	✓	Train
90	30	60	180		
105	105	105	315	✓	Train

Table S2. Fertilization trial (F) in 2019.

Nitrogen inputs (kgN/ha) at:			Total nitrogen inputs (kg.ha-1)	Destructive measurements	Train/Val
Tillering	Stem elongation	Flag leaf			
0	0	0	0	✓	Train
30	30	30	90	✓	Train
0	60	60	120		
60	60	0	120		
90	30	60	180		
60	60	60	180	✓	Train
80	40	60	180	✓	Val
105	105	105	315	✓	Train

Table S3. Trials composed of different fertilization fractioning combined with different fungicide application programs (FP) in 2020, 2021 and 2022.

Nitrogen inputs (kgN/ha) at:			Total nitrogen inputs (kg.ha-1)	Fungicide at BBCH stages	Destructive measurements	Train/Val
Tillering	Stem elongation	Flag leaf				
40	40	40	120	/	✓	Train
60	60	60	180	/		
80	60	60	200	/		
100	80	80	260	/	✓	Train
40	40	40	120	39	✓	Val
60	60	60	180	39	✓	Train
80	60	60	200	39		
100	80	80	260	39	✓	Val
40	40	40	120	32 - 55		
60	60	60	180	32 - 55		
100	80	80	260	32 - 55		
40	40	40	120	32 - 39 - 65	✓	Train
60	60	60	180	32 - 39 - 65		
80	60	60	200	32 - 39 - 65		
100	80	80	260	32 - 39 - 65	✓	Train

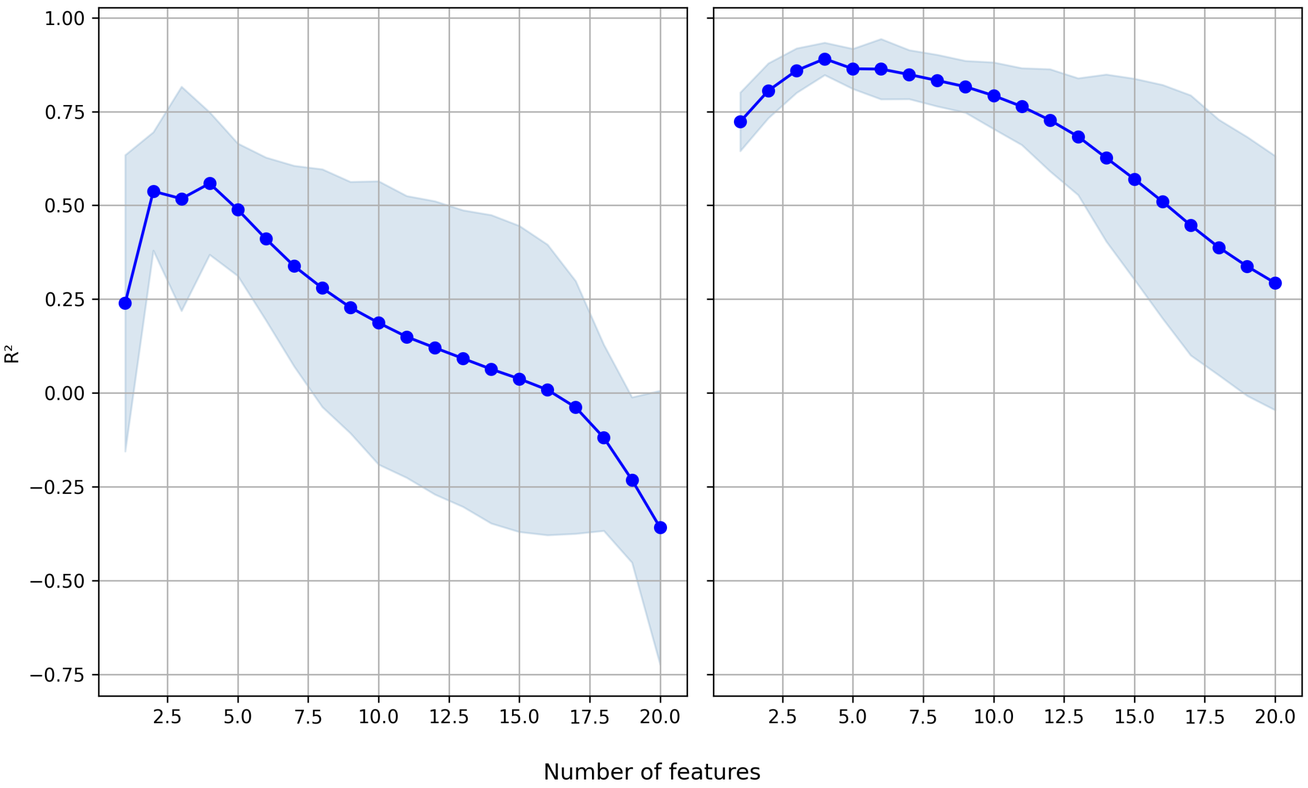

Figure S1. Backward feature selection with PLSr for DM Estimation: On the left, selected features for estimating DM from Ytrue include Plant Ratio, 95th Percentile of Height, MCARI, and BRF 490. On the right, the selected features for estimating DM from Ypseu comprise Plant Ratio, 95th Percentile of Height, MCARI, and BRF 550.

REFERENCES

Barnes, E. M., Clarke, T. R., Richards, S. E., Colaizzi, P. D., Haberland, J., Kostrzewski, M., et al. (2000). Coincident detection of crop water stress, nitrogen status and canopy density using ground-based multispectral data. In *Proceedings of the Fifth International Conference on Precision Agriculture* (Bloomington, Madison, USA)

Table S4. Dry matter, LAI, nitrogen concentration and nitrogen uptake of the whole plant used in this study. Data are means +/- the standard deviation (n=3 or 4 repetitions). Significance of treatment effects were analyzed using a one-way ANOVA (*: P $\leq$ 0.05; **: P $\leq$ 0.01; **:, P $\leq$ 0.001).

Trial	year	stage	Dry matter (T/ha)	LAI	N concentration (%)	N uptake (kg N/ha)
F	2019	30	1.36 +/- 0.31	1.65 +/- 0.40	3.37 +/- 0.76***	46.58 +/- 16.34**
		32	3.39 +/- 0.80***	3.68 +/- 1.15***	2.74 +/- 0.81***	96.18 +/- 39.63***
		39	6.77 +/- 1.30***	4.41 +/- 1.43***	1.73 +/- 0.50***	121.04 +/- 48.83***
		65	9.37 +/- 2.03**	4.06 +/- 1.75***	1.40 +/- 0.41***	137.75 +/- 62.79***
		75	13.75 +/- 3.05**	2.12 +/- 0.86***	1.15 +/- 0.30***	162.94 +/- 64.29***
		89	17.73 +/- 4.01**	0.00 +/- 0.00	1.01 +/- 0.22***	184.44 +/- 69.08***
	2020	32	5.01 +/- 0.91*	3.97 +/- 1.12**	2.49 +/- 0.60***	127.52 +/- 46.34***
		39	8.11 +/- 1.36***	5.72 +/- 1.75***	1.89 +/- 0.51***	157.23 +/- 55.55***
		65	12.75 +/- 1.54*	3.50 +/- 0.92***	1.32 +/- 0.29***	170.03 +/- 51.86***
		75	16.46 +/- 2.65***	3.22 +/- 0.98***	1.18 +/- 0.22***	197.95 +/- 58.29***
		89	18.91 +/- 2.49***	0.00 +/- 0.00	1.10 +/- 0.17***	210.40 +/- 51.27***
	2021	30	1.19 +/- 0.21*	1.48 +/- 0.35**	3.66 +/- 0.55***	44.25 +/- 12.29***
		32	3.98 +/- 0.73***	4.01 +/- 1.03***	2.40 +/- 0.47***	97.47 +/- 32.25***
		39	7.33 +/- 1.44**	5.00 +/- 1.78***	1.75 +/- 0.52***	131.98 +/- 54.17***
		65	10.85 +/- 1.63**	4.31 +/- 1.33***	1.48 +/- 0.37*	164.44 +/- 57.74**
		75	17.55 +/- 2.02**	2.46 +/- 0.80***	1.13 +/- 0.28***	201.83 +/- 67.94***
		89	17.03 +/- 3.23***	0.00 +/- 0.00	1.27 +/- 0.21**	218.50 +/- 60.96***
	2022	30	0.71 +/- 0.14*	1.09 +/- 0.24	3.57 +/- 0.42***	25.76 +/- 6.81**
		65	9.02 +/- 1.71**	3.20 +/- 0.88***	1.37 +/- 0.24	123.89 +/- 32.43**
		89	16.88 +/- 3.22***	0.00 +/- 0.00	1.34 +/- 0.23***	232.29 +/- 72.87***
FP	2020	38	8.20 +/- 1.50	3.53 +/- 0.92	1.89 +/- 0.32***	155.39 +/- 41.15*
		65	12.82 +/- 1.78	4.16 +/- 0.89	1.42 +/- 0.18***	183.78 +/- 42.32**
		89	19.35 +/- 1.55	0.00 +/- 0.00	1.19 +/- 0.14***	230.71 +/- 39.91**
	2021	39	8.70 +/- 2.48	5.44 +/- 1.70	1.67 +/- 0.29***	145.61 +/- 49.44
		65	13.33 +/- 1.74	4.61 +/- 1.04	1.56 +/- 0.25***	209.37 +/- 47.45
		89	17.93 +/- 1.91	0.00 +/- 0.00	1.33 +/- 0.21***	238.06 +/- 38.81
	2022	30	0.78 +/- 0.10	1.13 +/- 0.16	3.49 +/- 0.26	27.09 +/- 3.90
		32	3.34 +/- 0.48	3.12 +/- 0.52	2.32 +/- 0.28*	77.95 +/- 17.35*
		39	6.31 +/- 1.05	3.10 +/- 0.63**	1.63 +/- 0.14**	103.78 +/- 22.94**
		65	9.01 +/- 1.48*	2.97 +/- 0.70**	1.53 +/- 0.17*	137.53 +/- 27.01**
		73	12.89 +/- 2.29***	2.17 +/- 0.76***	1.44 +/- 0.29	185.99 +/- 52.06**
		89	15.19 +/- 3.24***	0.00 +/- 0.00	1.42 +/- 0.16	214.95 +/- 51.72**

Daughtry, C. S. T., Walthall, C. L., Kim, M. S., de Colstoun, E. B., and McMurtrey, J. E. (2000). Estimating Corn Leaf Chlorophyll Concentration from Leaf and Canopy Reflectance. *Remote Sensing of Environment* 74, 229–239. doi:10.1016/S0034-4257(00)00113-9

Devadas, R., Lamb, D. W., Simpfendorfer, S., and Backhouse, D. (2009). Evaluating ten spectral vegetation indices for identifying rust infection in individual wheat leaves. *Precision Agriculture* 10, 459–470. doi:10.1007/s11119-008-9100-2

Gitelson, A. A., Keydan, G. P., and Merzlyak, M. N. (2006). Three-band model for noninvasive estimation of chlorophyll, carotenoids, and anthocyanin contents in higher plant leaves. *Geophysical Research Letters* 33. doi:10.1029/2006GL026457

Haboudane, D., Miller, J. R., Tremblay, N., Zarco-Tejada, P. J., and Dextraze, L. (2002). Integrated narrow-band vegetation indices for prediction of crop chlorophyll content for application to precision

Table S5. R^2 of the different models to predict DM and Nupt proportion of each organ.

Model	Data	Dataset	Nuptake				DM			
			Stem	Linf	L1	Ear	Stem	Linf	L1	Ear
EfficienNetB0	Ypseu	train	0.95	0.99	0.75	0.91	0.59	0.87	-0.14	0.95
EfficienNetB0	Ytrue	train	0.82	0.93	0.61	0.88	0.91	0.96	-0.02	0.93
EfficienNetB4	Ypseu	train	0.49	0.68	0.65	0.62	0.58	0.87	-0.37	0.93
EfficienNetB4	Ytrue	train	0.66	0.83	-1.38	0.55	0.93	0.98	0.5	0.95
ResNet50	Ypseu	train	0.81	0.95	0.73	0.92	0.52	0.85	0.04	0.86
ResNet50	Ytrue	train	0.97	0.99	0.84	0.99	0.98	0.99	0.71	0.98
EfficienNetB0	Ypseu	val	0.83	0.97	0.75	0.87	0.58	0.87	-0.17	0.94
EfficienNetB0	Ytrue	val	0.77	0.91	0.49	0.85	0.84	0.95	-0.31	0.92
EfficienNetB4	Ypseu	val	0.4	0.7	0.49	0.51	0.62	0.88	-0.24	0.93
EfficienNetB4	Ytrue	val	0.55	0.83	-1.8	0.44	0.87	0.96	0.2	0.94
ResNet50	Ypseu	val	0.63	0.92	0.73	0.88	0.59	0.86	0.12	0.87
ResNet50	Ytrue	val	0.78	0.96	0.66	0.9	0.91	0.98	0.46	0.96

Table S6. Pearson correlation table of the agronomic data

Variables	Dry matter (T/ha)	LAI	N concentration (%)	N uptake (kg N/ha)
Dry matter (T/ha)	1.00	-0.27	-0.71	0.87
LAI	-0.27	1.00	0.16	0.03
N concentration (%)	-0.71	0.16	1.00	-0.44
N uptake (kg N/ha)	0.87	0.03	-0.44	1.00

Table S7. Vegetation indices selected in this study.

Name	VI formulation	Reference
NDRE	$\frac{BRF800-BRF720}{BRF800+BRF720}$	Barnes et al. (2000)
mNDb	$\frac{BRF490-BRF720}{BRF490+BRF800}$	Jay et al. (2017)
CIrede	$\frac{BRF800}{BRF720}-1$	Gitelson et al. (2006)
NDVI	$\frac{BRF800-BRF680}{BRF800+BRF680}$	Rouse et al. (1974)
PSRI	$\frac{BRF680-BRF490}{BRF800}$	Devadas et al. (2009)
SR	$\frac{BRF800}{BRF680}$	
GR	$\frac{BRF550}{BRF680}$	
GNDVI	$\frac{BRF800-BRF550}{BRF800+BRF550}$	Rouse et al. (1974)
OSAVI	$\frac{BRF800-BRF680}{BRF800+BRF680+0.16}$	Haboudane et al. (2002)
MSAVI	$\frac{2\cdot BRF800+1-\sqrt{(2\cdot BRF800+1)^2+8\cdot(BRF800-BRF680)}}{2}$	Qi et al. (1994)
MCARI	$\frac{(BRF720-BRF680)-0.2\cdot(BRF720-BRF550)}{BRF720/BRF680}$	Daughtry et al. (2000)
CIgreen	$\frac{BRF800}{BRF550}-1$	Gitelson et al. (2006)

agriculture. *Remote Sensing of Environment* 81, 416–426. doi:10.1016/S0034-4257(02)00018-4

Jay, S., Gorretta, N., Morel, J., Maupas, F., Bendoula, R., Rabatel, G., et al. (2017). Estimating leaf chlorophyll content in sugar beet canopies using millimeter- to centimeter-scale reflectance imagery. *Remote Sensing of Environment* 198, 173–186. doi:10.1016/j.rse.2017.06.008

Qi, J., Chehbouni, A., Huete, A. R., Kerr, Y. H., and Sorooshian, S. (1994). A modified soil adjusted vegetation index. *Remote Sensing of Environment* 48, 119–126. doi:10.1016/0034-4257(94)90134-1

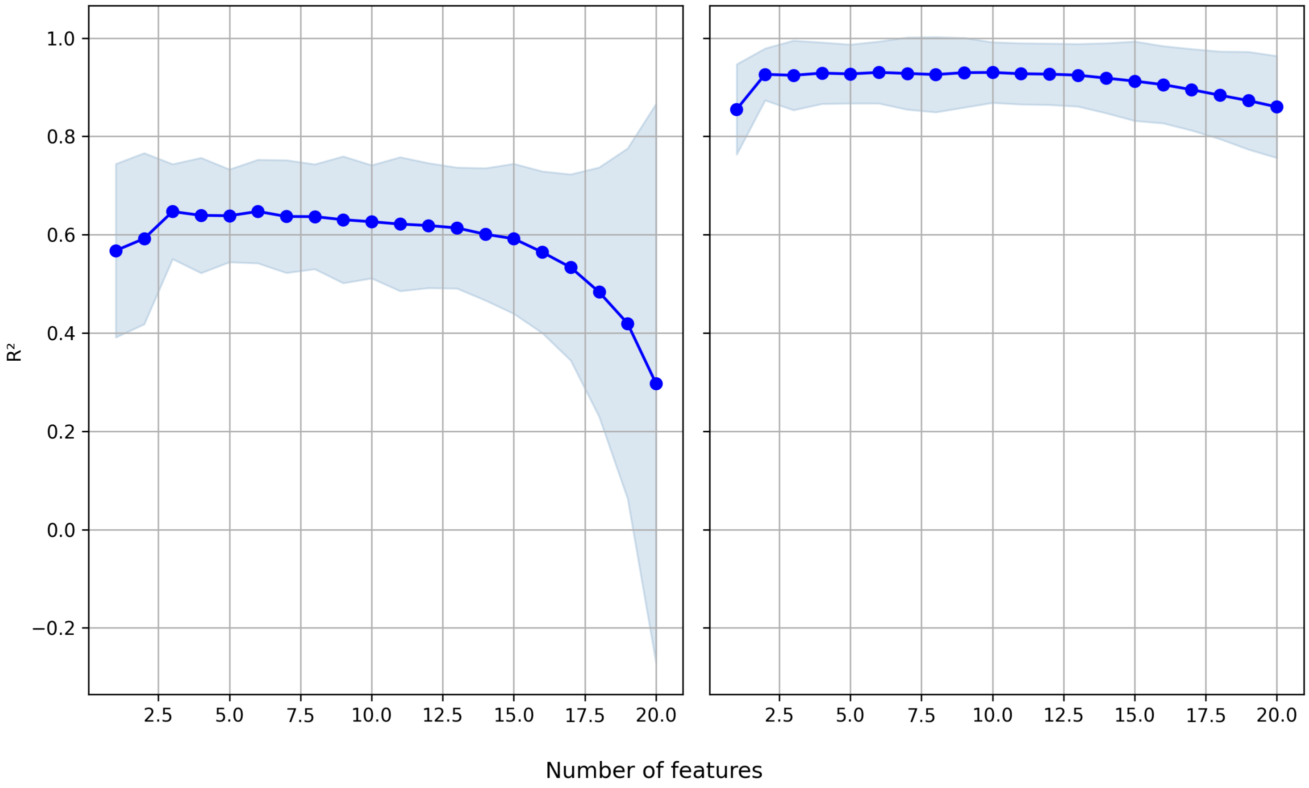

Figure S2. Backward feature selection with PLSr for LAI Estimation: On the left, selected features for estimating LAI from Ytrue include SR, GNDVI, MCARI, CIgree, BRF 900 and BRF 720. On the right, the selected features for estimating LAI from Ypseu comprise NDRE, SR, GNDVI, CIgreen, CIrede and BRF 550

Rouse, J. W., Haas, R. H., Schell, J. A., and Deering, D. W. (1974). Monitoring vegetation systems in the Great Plains with ERTS

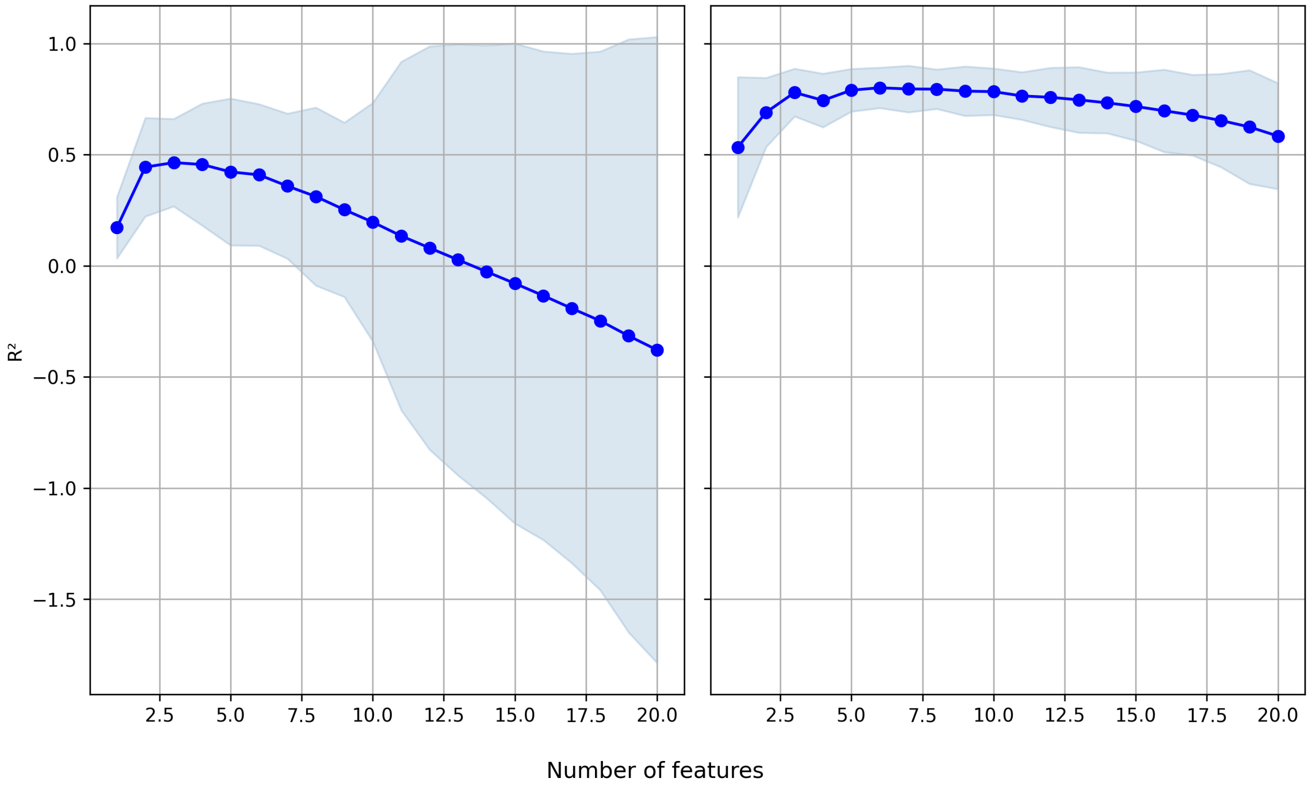

Figure S3. Backward feature selection with PLSr for Nuptake Estimation: On the left, selected features for estimating Nuptake from Ytrue include Plant Ratio, 95th percentile of height and MCARI. On the right, the selected features for estimating Nuptake from Ypseu comprise Plant Ratio, 95th percentile of height, GR, MCARI, mNDB and BRF 550.

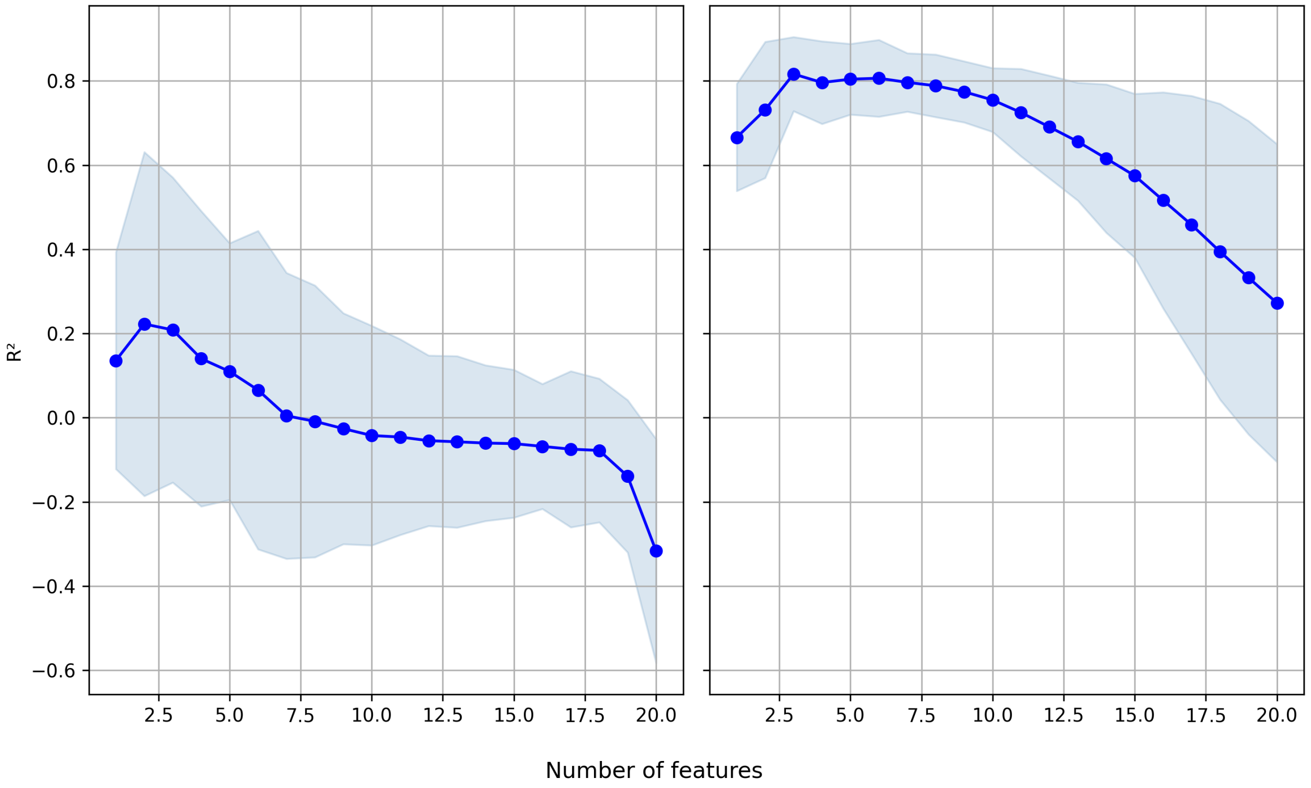

Figure S4. Backward feature selection with PLSr for Nrate Estimation: On the left, selected features for estimating Nrate from Ytrue include 95th percentile of height and MCARI.. On the right, the selected features for estimating Nrate from Ypseu comprise 95th percentile of height, GR and mNDB.
